# Threatened species richness along a Himalayan elevational gradient: quantifying the influences of human population density, range size, and geometric constraints

**DOI:** 10.1186/s12898-018-0162-3

**Published:** 2018-02-07

**Authors:** Prakash Kumar Paudel, Jan Sipos, Jedediah F. Brodie

**Affiliations:** 1 Center for Conservation Biology, Kathmandu Institute of Applied Sciences, PO Box 23002, Kathmandu, Nepal; 2 Department of Zoology, Fisheries, Hydrobiology and Apiculture, Mendel University in Brno, Brno, Czech Republic; 3 Department of Vegetation Ecology, Institute of Botany, The Czech Academy of Sciences, Brno, Czech Republic; 4 Division of Biological Sciences and Wildlife Biology Program, University of Montana, Missoula, MT USA

**Keywords:** Biodiversity conservation, Elevational gradient, Himalaya, Nepal, Threatened species

## Abstract

**Background:**

A crucial step in conserving biodiversity is to identify the distributions of threatened species and the factors associated with species threat status. In the biodiversity hotspot of the Himalaya, very little is known about which locations harbour the highest diversity of threatened species and whether diversity of such species is related to area, mid-domain effects (MDE), range size, or human density. In this study, we assessed the drivers of variation in richness of threatened birds, mammals, reptiles, actinopterygii, and amphibians along an elevational gradient in Nepal Himalaya.

**Results:**

Although geometric constraints (MDE), species range size, and human population density were significantly related to threatened species richness, the interaction between range size and human population density was of greater importance. Threatened species richness was positively associated with human population density and negatively associated with range size.

**Conclusions:**

In areas with high richness of threatened species, species ranges tend to be small. The preponderance of species at risk of extinction at low elevations in the subtropical biodiversity hotspot could be due to the double impact of smaller range sizes and higher human density.

**Electronic supplementary material:**

The online version of this article (10.1186/s12898-018-0162-3) contains supplementary material, which is available to authorized users.

## Background

Maintaining conservation efforts for all species in all areas simultaneously is not feasible due to limited resources [1]. Therefore, scientists often attempt to identify particularly biodiverse regions on which to focus conservation efforts [2]. Many of the approaches to identify such areas assess vulnerable taxa or vulnerable ecosystem types as a basis for prioritization [3]. The World Conservation Union (IUCN) Red List categories and criteria provide objective and quantitative frameworks for classifying the risk of global extinction [4, 5]. Threat status is known to be influenced by ecological factors such as population size, range size, and trends in abundance [4], as well as by human-induced impacts [6, 7].

Aside from knowing which species are threatened and why, it is also important to understand where threatened species are distributed. But biogeographical patterns in the distribution of threatened species have received relatively little attention. Hotspots of extinction risk tend to be in areas with high human population density or heavy anthropogenic impacts [8]. Many are also in low elevations. This could be due, in part, to range size distribution patterns whereby species ranges tend to be smaller at low elevations, and shifts in elevation range result in shrinking habitatable area [9]. Thus, range size is known to have a strong, negative influence on extinction risk [4]. Elevation is a critical biogeographical gradient in many parts of the world, and species richness patterns may be either linear or hump-shaped with respect to elevation [10–16]. Geographical area is positively associated with species diversity, and area decreases gradually with increasing elevations. Therefore, there tend to be fewer species at high elevations in mountainous regions [17]. Similarly, it has also been argued that mid-elevation peaks in diversity are inevitable due to geographical constraints known as mid-domain effects (MDE) [13, 15]. Theory suggests that hump-shaped relationships between diversity and elevation arise from random placement of species geographical ranges between hard boundaries (i.e., mountain tops and valley bottoms) [12, 13]. Some studies have shown that MDEs explain most of the variability in species richness [13, 15, 18–21]; while other studies have found that they account for little or no variation in species richness [22, 23]. However, the influences of anthropogenic factors such as human population density on elevational gradients in species richness have been surprisingly neglected, despite a large literature suggesting that biodiversity-rich areas often overlap with regions of high human density because both co-occur in the highly productive areas [24–26].

We examined the biogeographical distribution of threat status, range sizes, and human population pressure in a biodiversity hotspot, the Himalayan Mountains of Nepal. Numerous studies on elevational gradients of species diversity in the Himalayas have focused on richness patterns of specific taxa (e.g., [11, 16, 25, 27]) or on identifying climatic factors correlated with variation in species richness [10, 14]. However, how species richness across multiple groups of threatened species responds to biogeographical variation in elevation, human influence, and range size have not been documented. Such information could contribute significantly to conservation planning and prioritization [28–30]. We use data on all of the threatened animal species found in Nepal to: (i) assess threatened species richness along elevational gradients, (ii) examine the effects of area, mid-domain effect (MDE), range size, and human population density on threatened species richness, and (iii) specify which elevation zone should receive the highest priority for conservation measures in the Nepal Himalayas.

## Methods

### Study region

**Fig. 1:**
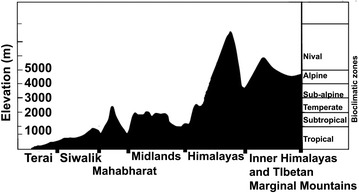
A cross-section of Nepal showing bioclimatic and physiographic zones [31]

Nepal is a mountainous country in the central Himalaya (26°22′–30°27′ N, 80°4′–88°12′ E) (Fig. 1a). It has three distinct mountain ranges with extreme variations in elevations (60–8848 m) over short horizontal distances (~ 200 km), generating a complex mosaic of habitats and ecological zones ranging from subtropical forests to alpine pastures [31] (Fig. 1). Due to the highly complex topography and variation in elevation, Nepal (0.1% of the global land mass) contains a disproportionately high diversity of plants and animals (~ 2% of the flowering plants, 4.5% of the pteridophytes, 3.8% of the mammals and 8.6% of the birds found globally [31]). However, this rich biodiversity is highly affected by human activities [32].

## Data

### Species data

We used a list of globally threatened species (i.e., critically endangered, endangered, and vulnerable) that occur in Nepal from the IUCN Red List of Threatened Species (http://www.iucnredlist.org), the most comprehensive database of the global conservation status of plants and animals [5]. Elevational range of each threatened species was obtained from a variety of sources (Additional file 1: List of threatened species). We excluded threatened species reported as extinct in Nepal and species with undetermined elevational ranges. We prepared a list of 71 threatened species (Mammalia—28 species, Reptilia—4 species, Aves—32 species, Amphibia—3 species, Actinopterygii—4 species) that have well-defined distribution ranges in Nepal for the data analysis.

### Data on species richness, human population density, range size, and geometric constraints

To quantify influence of human population density, range size, and geometric constraints on threatened species richness along a Himalayan elevational gradient, we used a set of five variables (Table 1). To derive species richness in each elevation gradient, we first divided the elevation gradient in the Nepal Himalaya (0–4900 m) into 49 zones of 100 vertical meters each. Elevation zones above 5000 m in Nepal are considered to be an arctic desert [31]. Climatic gradients in Nepal suggest that conditions change markedly every 100 m vertical distance [33], and this elevational bin is standard for analyses of the diversity of a variety of taxa in Nepal [11, 14, 16, 27, 33]. A species was assumed to be present in each 100 m interval between its upper and lower elevational limits. We computed species richness of each zone (elevational band) by interpolating ranges of the 71 threatened species in our database. Such approaches are well-established in the assessment of elevational gradients of species diversity [10, 11, 16, 27].

**Table 1:** List of variables used in the model

Sn	Variables	Descriptions
1	Species richness	Number of threatened species found in each elevational band
2	Null model	Species richness predicted for each elevational band from the mid-domain effect null model
3	Elevation	Elevation band along an altitudinal gradient (100–4900 m asl)
4	Range size	The difference between lower and upper distributional limits of a species in an altitudinal gradient
5	Population density	Human population density estimated for each elevational band

Interpolation method helps in overcoming problems of undersampling [16, 34]. However, we also explicitly examined potential influences of undersampling following the methodology described in [34], with minor modifications. Interpolation methods assume that a species is present between its upper and lower elevation limits. The lack of systematic sampling may erroneously underestimate ranges of species. The problem is particularly acute for species with small ranges. We first categorized species into three classes: (a) range size less than 1300 m (> 26% of gradient), (b) range size between 1300 and 2550 m (26–51% of gradient), and (c) range size greater than 2550 m (> 51% of gradient). We then augmented the range sizes of species for three different scenarios based on range-size-specific rules, assuming a decreasing probability of error with increasing range size: [20, 10, 0%], [30, 20, 10%], [50, 25, 10%]. For example, range sizes of species were augmented by 20% of the gradient (1000 m) for category ‘a’ species, by 10% of the gradient (500 m) for category ‘b’ species, and by 0% of range (no change) for category ‘c’ species. The same method was applied for the other two scenarios: ([30, 20, 10%], [50, 25, 10%]). If a species, for example, has elevation range between 200 and 1400 m (range size 1200 m), we augmented the range, according to the first augmentation rule, to have a lower limit of 67 m and an upper limit of 1900 m because lower or upper elevational limits are constrained between 67 and 4900 m. We tested whether there was an influence of interpolation and under-sampling in our data using Pearson correlation tests. We adjusted for multiple comparisons by using Bonferroni corrections among species richness patterns, based on the three augmentation scenarios and empirically measured richness using a “corr.test” function in the psych package in R statistical software (R Development Core Team, 2015). The test showed that there was a significant correlation in species richness patterns between the empirical data and the first augmentation (r = 0.72, *P* < 0.001), and between the empirical data and the second augmentation (r = 0.38, *P* = 0.02). However, the correlation in species richness patterns was not significant between the empirical data and the third augmentation (r = 0.21, *P* = 0.15). We constructed scatter plots between species richness generated by augmented range sizes and exploratory variables, which suggested similar trends among the exploratory variables in all augmentation scenarios (Additional file 2: Plot S1).

We defined range size of a species as the difference between lower and upper distributional limits. For example, if a species has range limit between 660 and 1000 m, its equivalent distribution in our dataset spans between the 600 and 1000 m elevational bins. Here, the range size of this species is 400 m and the mid-point is 800 m (average of lower and upper elevational bins). Thus, the species is assumed to be present in the elevation zone of 800 m (between 700 and 800) with a 400 m range size. Range sizes of each elevational band were calculated as averages of the ranges of all species in that band.

Human population count data at the ward level (the smallest administrative unit of Nepal; average area = 4.35 km^2^) was obtained from the Central Bureau of Statistics, Government of Nepal [35]. The data were mapped as the centroid of the ward for all municipalities and villages. We applied a kernel density transformation to the point feature data, which uses a quadratic kernel function to visualize human population density (individuals per km^2^) [36].

### Data analysis

We assessed a potential mid-domain peak in diversity, or a mid-domain effect (MDE), using a null model. We ran 5000 Monte Carlo simulations of empirical range sizes, without replacement, using the Microsoft Excel Add-in “Mid-domain null” [37]. Empirical midpoints were randomized across elevations [37]. This provides a simple, non-biological explanation for mid-elevation peaks in species richness.

We first tested for multicollinearity among the variables (Table 1) by calculating variance inflation factors (VIF). A VIF value greater than 10 is regarded as severe multicollinearity [38]. VIF analysis suggested that no pairs of the six input variables were problematically correlated (Table 2). We then used generalized least square (GLS) models, which account for potential autocorrelation [39], to quantify the influences of human population density, range size, and geometric constraints on threatened species richness. We used generalized additive (GAM) and generalized linear models (GLM) with cubic regression splines for graphical visualization of the data because the response curves were not constrained. This makes it easier to model complex predictor–response relationships.

**Table 2:** Summary of variance inflation factor calculated from the results of multiple regression model

Variable	Variance inflation factors
Null model	1.566
Elevation	3.532
Range sizes	4.478
Population density	2.944
Range sizes: population density	3.478

We fitted the relationships between threatened species richness and explanatory variables (area, MDE, elevation, population density, and species range sizes) using marginal mixed models on the basis of generalized least square methods. We used the ‘gls’ function of the ‘nlme’ package in R [40]. Dependant variables were log-transformed to normalize and homogenize residuals. We identified spatial autocorrelation in our data by using the “variogram” function. Spatial autocorrelation was accounted for by defining a residual variance–covariance matrix using the spatial correlation function “corGaus” of the “nlme” package [40]. We compared models with different spatial correlation functions of residuals using Akaike’s information criterion (AIC). The model with the lowest AIC value was chosen as the best model. In the next step, we tested the effect of explanatory variables by the likelihood-ratio test using a Chi square statistics. The likelihood-ratio is a test that compares the goodness of fit of two nested models, where a simple model is a special case of the complex model.

We used GAM and GLM models with Poisson error distributions, and controlled for over-dispersion, to assess the combined effects of population density and range sizes as well as the individual effects of the rest of explanatory variables on species richness. The GLM was used to show species richness along an altitudinal gradient where we used residuals of the GLM—obtained after removing the effect of area and the mid-domain effect in the model—as the dependent variable.

In the GLM and GAM models, we tested the effects of explanatory variables by analysis of deviance (ANODEV) with sequential sums of squares. We ordered area, MDE, and elevation as the first three explanatory variables in ANODEV to control for spatial autocorrelation and geographical area. These analyses were performed in R.

## Results

**Fig. 2:**
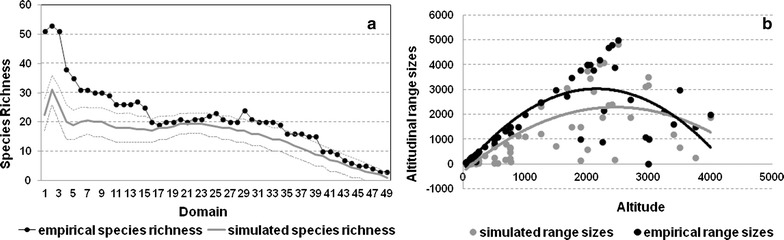
**a** A null model developed by 5000 Monte Carlo simulations (without replacement) of empirical range size (dark solid line) [37]. Plotted line with dark markers depicts the empirical species richness. **b** Second order polynomial regression between simulated range size (y = 0.00x^2^ + 2.10x − 296.80, R^2^ = 0.64, P < 0.001) and empirical range size (y = 0.00x^2^ + 2.30x − 195.48, R^2^ = 0.56, P < 0.001). Simulated range sizes are plotted on the secondary axis

A null model produced by Monte Carlo permutations of empirical range size (without replacement) produced a monotonically decreasing pattern of species richness with elevation (Fig. 2a). There was a significant correlation between simulated range sizes and empirical range sizes (Spearman’s rank correlation, *P* < 0.001, R^2^ = 0.60; Fig. 2b), and between threatened species richness and null model estimates (Table 3).

**Table 3:** Summary of analysis of deviance (ANODEV) of generalized additive model with threatened species richness regressed against a null model (“bs” representing cubic regression spline) (R^2^ = 0.17)

	Df	Deviance	Residual Df	Residual deviance	F value	P value
Null			34	99.626		
bs (null.model)	3	38.992	31	60.634	6.819	< 0.001

**Fig. 3:**
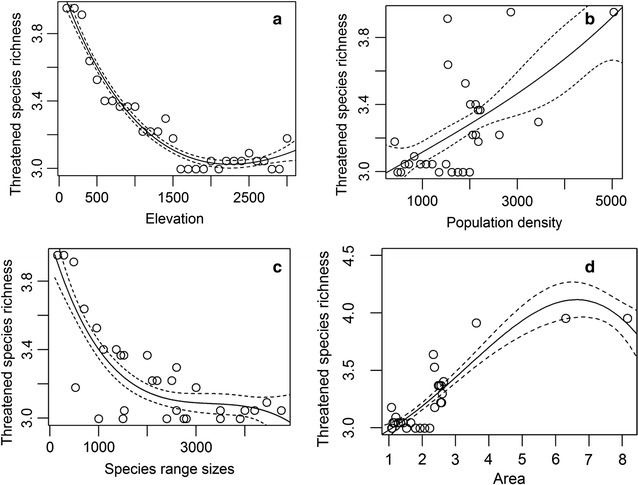
Relationship between threatened species richness with **a** elevation; **b** population density; **c** species range sizes and **d** area and along 100-m zone elevational gradient in Nepal Himalaya. The dependent variable, threatened species richness, was log transformed. The solid line represents a cubic regression spline fitted by GAM. Dashed lines represent standard errors

**Fig. 4:**
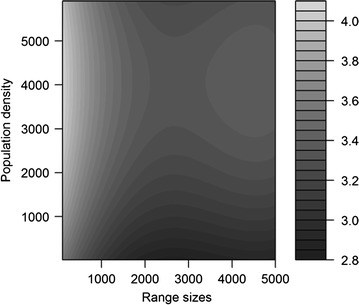
Effects on threatened species richness by human population density (Chisq_3, 28_ = 19.24, P < 0.01, R^2^ = 0.47) and range size (Chisq_3, 31_ = 45.60, P < 0.001, R^2^ = 0.69). The colour shade indicates species richness—the lighter the shade the higher the richness. Values of species richness were predicted by the generalized additive model in which population density and range sizes were transformed by cubic regression spline

**Fig. 5:**
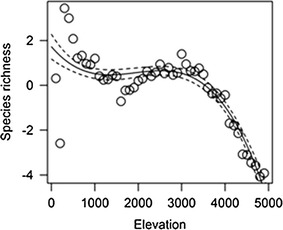
Relationship between threatened species richness and elevation after controlling the influence of geographical area and mid-domain effects. The solid line was fitted by a generalized liner model (y = − 6.16x^3^ + 3.01x^2^ − 3.24x + 1.51, R^2^ = 0.73, P = 0.017). (Note that the vertical axis has different scales.) Dashed lines represent standard errors

Threatened species richness showed an overall decreasing trend along the elevational gradient; richness was high at low elevations, decreased with increasing elevation up to ~ 2000 m, and became flat above that (Fig. 3a). Here, species richness showed a positive relationship with human population density (Fig. 3b) and area of the elevational band (Fig. 3d). Threatened species richness was negatively associated with species range sizes (Fig. 3c). Species with relatively small elevational ranges contributed to the peak of species richness, which occurred in the most densely populated locations (Fig. 4). When the influence of geographical area and mid-domain effects were controlled for (i.e., residuals were used as dependant variables), threatened species richness along the elevation gradient showed a low plateau pattern whereby richness increased gradually to a peak at ~ 3500 m (Table 4, Fig. 5). Thus, modeled total richness was bimodal, peaking at < 200 m and at 2500–3500 m. Observed richness was also bimodal, peaking at ~ 300 and ~ 3100 m (Fig. 5). A generalized additive model suggested that MDEs significantly affected threatened species richness pattern (Table 3). After the model was controlled for spatial autocorrelation (i.e. using generalized least squares) threatened species richness was significantly affected only by area and by the interaction between species range size and human population density (Table 5).

**Table 4:** Results of analysis of deviance (ANODEV) of generalized linear model with residuals regressed against altitude

	Df	Deviance	Residual Df	Residual deviance	F value	P value
Null	1		34	16.451		
Poly (elevation, 3)	3	4.511	31	11.940	3.904	0.017

Residuals were obtained after removing effect of area and mid-domain effect in the model. Function “poly” represents a cubic transformation of the explanatory variable. This function also contains both linear and polynomial effects

**Table 5:** Results of likelihood-ratio analysis of deviance of marginal mixed models using generalized least square with threatened species richness as a dependent variable

	Df	AIC	LRT	P value
(Full model)		− 50.202		
Area	1	− 44.958	7.244	< 0.01
Null model (simulated range)	1	− 51.967	0.235	0.627
Elevation	1	− 48.772	3.430	0.063
Empirical range: population density	1	− 48.078	4.124	0.042

Likelihood-ratio analysis tested whether the deviance of the full model significantly increases after each explanatory variable was excluded from the model. The denominator Df = 28

## Discussion

Understanding elevational patterns of species richness, and their underlying mechanisms, are critically important for conservation biology. Such studies, however, have been contentious due to the lack of consistent results across taxa and under different biophysical conditions. Some studies suggest that species richness peaks at intermediate elevations [37, 41] whereas others report that species richness decreases with increasing elevation [42]. In our study, richness of threatened species of birds, mammals, reptiles, actinopterygii, and amphibians exhibited a monotonically decreasing trend along a Himalayan elevation gradient. Such a pattern contrasts to those identified for plants (trees, ferns, lichens, orchids, and liverworts) [10, 11, 27] in the Nepal Himalaya.

The area of a habitat patch (or elevational band, in our case) is one of the strongest determinants of the species richness (Table 5). This is in agreement with the habitat diversity hypothesis [17, 43, 44]. According to this hypothesis, large areas contain more species because they have higher habitat heterogeneity that provides more niches [17, 45]. After controlling for the effect of area, species richness declined, on average, with increasing elevation, but with peaks at relatively low (100–300 m) and intermediate (2500–3500 m) elevations—essentially exhibiting a low plateau pattern (Fig. 5). Similar patterns have been observed for birds in the Himalayas [16], but most other taxa (e.g., ferns, trees, and mosses) have unimodal patterns with peak of species diversity at intermediate elevations [46]. McCain [47] analysed elevational gradients for small mammals and found that gamma diversity was influenced by area, with a trend of highest diversity at lower elevations, similar to our findings.

Mid-elevation peaks are sometimes regarded as sampling artefacts because random placement of large ranges produces an artificial peak in the middle of the gradient [13]. Our study indicated that geometric constraint (i.e., a mid-domain effect) influenced threatened species richness (Table 3). However, it had less explanatory power than the human population density and range size in our analysis (Table 5). With increasing human population, the threatened species richness increased steeply (Fig. 3b). This is consistent with other studies that have shown that biodiversity-rich regions overlap with areas densely settled by humans [8, 24]. In our study, threatened species richness was negatively associated with range sizes (Fig. 3c). Range size along elevational gradients is determined by intrinsic ecological and life-history traits, as well as anthropogenic activities, which increases the extinction risk [8, 48].

That highest richness of threatened species occurred in densely populated areas in our study region suggests important conservation message. Species with small range sizes are likely to have smaller niche breaths [49] and may further face range shrinkages due to climate change [9, 50] and human induced habitat modifications [9]. This is consistent with the findings in extinction patterns in carnivores, where intrinsic factors were critical determinants of risk that increased synergistically as human populations expanded [8]. Therefore, human pressure on threatened species should not be underestimated in light of the positive association between population density and threatened species richness (Fig. 3b).

## Conclusions and implications for conservation

To date, no study of this kind has focused on threatened species in the Himalaya. Our analyses are based on high-resolution data of national distributions of birds, mammals, reptiles, actinopterygii, and amphibians. We identify regions of high threatened species richness based on multiple taxonomic groups, and suggest that conservation attention is urgently needed in these areas.

Our results show that both lowlands (300–400 m) and midlands (2500–3500 m) are biodiversity hotspots in Nepal, with disproportionate representation of threatened species. Areas below 400 m in Nepal are densely settled by humans, supporting more than 45% of its 2.3 million people [51]. Therefore, habitat available in these elevation zones may be limited. Forests between 300 and 400 m elevation, despite being highly fragmented, are better protected by reserves than those at lower elevations, and may serve as refuges for lowland species [31, 52]. Forest remnants in these elevational zones may therefore be critical for the conservation of Nepal’s threatened species as there is no longer space to expand reserves in lower elevation areas. At intermediate elevations, many of the habitat patches are forested islands on mountain peaks, surrounded by human-exploited landscapes (Paudel, unpublished data). Such inhospitable intervening habitat matrix can increase the sensitivity of species richness or occurrence to patch area and isolation.

We also show that species with small ranges contributed to the diversity peaks in these hotspots. This could have implications for conservation planning in the face of climate change. For example, species that are shifting their elevation in response to changing abiotic conditions may be trapped in montane islands at intermediate elevations. Species distributed over small ranges will be particularly vulnerable [49, 53]. Expansion of protected areas in Nepal, particularly at lower and middle elevations, is critical to reduce the impact of human activities on a biota of global significance.

## Additional files

**Additional file 1.** List of threatened species. Threatened species (Endangered, Vulnerable and Critically Endangered) found in Nepal according to IUCN Red List of Threatened Species. Version 2014.2.

**Additional file 2: Plot S1.** Correlation of population density, area, species range size and elevation with species richness patterns measured with empirical data and three augmentation scenarios.

## References

[1] Sarkar S, Pressey RL, Faith DP, Margules CR, Fuller T, Stoms DM (2006). Biodiversity conservation planning tools: present status and challenges for the future. Annu Rev Environ Resour.

[2] Margules CR, Pressey RL (2000). Systematic conservation planning. Nature.

[3] Myers N, Mittermeier RA, Mittermeier CG, da Fonseca GAB, Kent J (2000). Biodiversity hotspots for conservation priorities. Nature.

[4] Gärdenfors U, Hilton-Taylor C, Mace GM, Rodríguez JP (2001). The application of IUCN Red List criteria at regional levels. Conserv Biol.

[5] IUCN. IUCN 2014. The IUCN Red List of threatened species. Version 2014.2. IUCN; 2014. http://www.iucnredlist.org. Accessed 24 July 2014.

[6] Purvis A, Gittleman JL, Cowlishaw G, Mace GM (2000). Predicting extinction risk in declining species. Proc R Soc Lond B Biol Sci.

[7] Harcourt AH, Parks SA (2003). Threatened primates experience high human densities: adding an index of threat to the IUCN Red List criteria. Biol Conserv.

[8] Cardillo M, Purvis A, Sechrest W, Gittleman JL, Bielby J, Mace GM (2004). Human population density and extinction risk in the world’s carnivores. PLoS Biol.

[9] Wilson RJ, Gutiérrez D, Gutiérrez J, Martínez D, Agudo R, Monserrat VJ (2005). Changes to the elevational limits and extent of species ranges associated with climate change. Ecol Lett.

[10] Acharya KP, Vetaas OR, Birks HJB (2011). Orchid species richness along Himalayan elevational gradients. J Biogeogr.

[11] Baniya CB, Solhøy T, Gauslaa Y, Palmer MW (2010). The elevation gradient of lichen species richness in Nepal. Lichenologist.

[12] Brehm G, Colwell RK, Kluge J (2007). The role of environment and mid-domain effect on moth species richness along a tropical elevational gradient. Glob Ecol Biogeogr.

[13] Colwell RK, Lees DC (2000). The mid-domain effect: geometric constraints on the geography of species richness. Trends Ecol Evol.

[14] Grau O, Grytnes J-A, Birks HJB (2007). A comparison of altitudinal species richness patterns of bryophytes with other plant groups in Nepal, Central Himalaya. J Biogeogr.

[15] Jetz W, Rahbek C (2001). Geometric constraints explain much of the species richness pattern in African birds. Proc Natl Acad Sci.

[16] Paudel PK, Sipos J (2014). Conservation status affects elevational gradient in bird diversity in the Himalaya: a new perspective. Glob Ecol Conserv.

[17] MacArthur RH, Wilson EO (1967). The theory of island biogeography.

[18] Hawkins BA, Diniz-Filho JAF (2002). The mid-domain effect cannot explain the diversity gradient of Nearctic birds. Glob Ecol Biogeogr.

[19] Cardelús CL, Colwell RK, Watkins JE (2006). Vascular epiphyte distribution patterns: explaining the mid-elevation richness peak. J Ecol.

[20] Kluge J, Kessler M, Dunn RR (2006). What drives elevational patterns of diversity? A test of geometric constraints, climate and species pool effects for pteridophytes on an elevational gradient in Costa Rica. Glob Ecol Biogeogr.

[21] McCain CM, Grytnes J-A. Elevational gradients in species richness. In: Wiley, editor. Encycl. Life Sci. Chichester: Wiley; 2010. http://www.els.net/WileyCDA/ElsArticle/refId-a0022548.html. Accessed 7 Dec 2014.

[22] Diniz-Filho JAF, De Sant’Ana CER, De Souza MC, Rangel TFLVB (2002). Null models and spatial patterns of species richness in South American birds of prey. Ecol Lett.

[23] Zapata FA, Gaston KJ, Chown SL (2005). The mid-domain effect revisited. Am Nat.

[24] Pimm SL, Russell GJ, Gittleman JL, Brooks TM (1995). The future of biodiversity. Sci AAAS Wkly Pap Ed.

[25] Cincotta RP, Wisnewski J, Engelman R (2000). Human population in the biodiversity hotspots. Nature.

[26] Luck GW (2007). A review of the relationships between human population density and biodiversity. Biol Rev.

[27] Bhattarai KR, Vetaas OR, Grytnes JA (2004). Fern species richness along a central Himalayan elevational gradient, Nepal. J Biogeogr.

[28] Gaston KJ (2000). Global patterns in biodiversity. Nature.

[29] Gaston KJ, Blackburn TM (1996). The spatial distribution of threatened species: macroscales and new world birds. Proc R Soc Lond B Biol Sci.

[30] Paudel PK, Heinen JT (2015). Think globally, act locally: on the status of the threatened fauna in the Central Himalaya of Nepal. Geoforum.

[31] Paudel PK, Bhattarai BP, Kindlmann P. An overview of the biodiversity in nepal. In: Kindlmann P, editor. Himal. Biodivers. Chang. World. Dordrecht: Springer Netherlands; 2012. p. 1–40. Available from: http://link.springer.com/chapter/10.1007/978-94-007-1802-9_1. Accessed 10 Nov 2014.

[32] Primack RB, Paudel PK, Bhattarai BP (2013). Conservation biology: a primer for Nepal.

[33] Bhattarai KR, Vetaas OR (2003). Variation in plant species richness of different life forms along a subtropical elevation gradient in the Himalayas, east Nepal. Glob Ecol Biogeogr.

[34] McCain CM (2007). Could temperature and water availability drive elevational species richness patterns? A global case study for bats. Glob Ecol Biogeogr.

[35] CBS N. National population and housing census 2011. Natl. Rep. 2012.

[36] Silverman BW (1992). Density estimation for statistics and data analysis.

[37] McCain CM (2004). The mid-domain effect applied to elevational gradients: species richness of small mammals in Costa Rica. J Biogeogr.

[38] Montgomery DC, Peck EA (1992). Introduction to Linear regression analysis.

[39] Pinheiro J, Bates DM (2000). Mixed-effects models in S and S-plus.

[40] Pinheiro J, Bates D, DebRoy S, Sarkar D. The R core team: nlme: linear and nonlinear mixed effects models. R package version 3.1 90. 2008.

[41] Wu Y, Colwell RK, Rahbek C, Zhang C, Quan Q, Wang C (2013). Explaining the species richness of birds along a subtropical elevational gradient in the Hengduan Mountains. J Biogeogr.

[42] Ohsawa M, Shakya PR, Numata M (1986). Distribution and succession of west Himalayan forest types in the eastern part of the Nepal Himalaya. Mt Res Dev.

[43] Connor EF, McCoy ED (1979). The statistics and biology of the species–area relationship. Am Nat.

[44] MacArthur RH (1984). Geographical ecology: patterns in the distribution of species.

[45] Shmida AVI, Wilson MV (1985). Biological determinants of species diversity. J Biogeogr.

[46] Paudel PK (2013). Conservation biology of the Himalayas. Conserv Sci.

[47] McCain CM (2005). Elevational gradients in diversity of small mammals. Ecology.

[48] Cooper N, Bielby J, Thomas GH, Purvis A (2008). Macroecology and extinction risk correlates of frogs. Glob Ecol Biogeogr.

[49] Harris G, Pimm SL (2008). Range size and extinction risk in forest birds. Conserv Biol.

[50] Sekercioglu CH, Schneider SH, Fay JP, Loarie SR (2008). Climate change, elevational range shifts, and bird extinctions. Conserv Biol.

[51] Government of Nepal. Nepal population report 11. Central Bureau of Statistics; 2011.

[52] Paudel PK, Heinen JT (2015). Conservation planning in the Nepal Himalayas: effectively (re)designing reserves for heterogeneous landscapes. Appl Geogr.

[53] Chen I-C, Hill JK, Ohlemüller R, Roy DB, Thomas CD (2011). Rapid range shifts of species associated with high levels of climate warming. Science.

