# Supplementary material for: Threatened species richness along a Himalayan elevational gradient: quantifying the influences of human population density, range size, and geometric constraints
Source: BMC Ecol. 2018 Feb 7;18:6. doi: 10.1186/s12898-018-0162-3 (PMC5803900; doi:10.1186/s12898-018-0162-3)
Supplement: Supplementary file 1 — Additional file 1. List of threatened species. Threatened species (Endangered, Vulnerable and Critically Endangered) found in Nepal according to IUCN Red List of Threatened Species. Version 2014.2. [file 12898_2018_162_MOESM1_ESM.docx]

| Additional file 1: List of threatened species  Threatened species (Critically Endangered, Endangered and Vulnerable) found in Nepal according to *The IUCN Red List of Threatened Species. Version 2014.2* | | | |
| --- | --- | --- | --- |
| Class | *Species* | Red List status* | Data sources |
| MAMMALIA | *Ailurus fulgens* | VU | [1,2] |
| MAMMALIA | *Aonyx cinerea* | VU | [1,3] |
| MAMMALIA | *Apodemus gurkha* | EN | [1] |
| MAMMALIA | *Arctictis binturong* | VU | [1] |
| MAMMALIA | *Axis porcinus* | EN | [1] |
| MAMMALIA | *Bos gaurus* | VU | [1,4] |
| MAMMALIA | *Bubalus arnee* | EN | [1,5] |
| MAMMALIA | *Caprolagus hispidus* | EN | [1] |
| MAMMALIA | *Cuon alpinus* | EN | [1,6] |
| MAMMALIA | *Elephas maximus* | EN | [1,7] |
| MAMMALIA | *Lutrogale perspicillata* | VU | [1,3] |
| MAMMALIA | *Manis pentadactyla* | EN | [1,8,9] |
| MAMMALIA | *Melursus ursinus* | VU | [1,10] |
| MAMMALIA | *Moschus chrysogaster* | EN | [1] |
| MAMMALIA | *Moschus Fuscus* | EN | [1] |
| MAMMALIA | *Moschus leucogaster* | EN | [1] |
| MAMMALIA | *Myotis sicarius* | VU | [1,11] |
| MAMMALIA | *Neofelis nebulosa* | VU | [1,12] |
| MAMMALIA | *Panthera tigris* | EN | [1,13] |
| MAMMALIA | *Panthera uncia* | EN | [1,14] |
| MAMMALIA | *Pardofelis marmorata* | VU | [1] |
| MAMMALIA | *Platanista gangetica* | EN | [1,15] |
| MAMMALIA | *Prionailurus viverrinus* | EN | [1] |
| MAMMALIA | *Rhinoceros unicornis* | VU | [1,16] |
| MAMMALIA | *Rucervus duvaucelii* | VU | [1] |
| MAMMALIA | *Rusa unicolor* | VU | [1] |
| MAMMALIA | *Tetracerus quadricornis* | VU | [1] |
| MAMMALIA | *Ursus thibetanus* | VU | [1] |
| REPTILIA | *Crocodylus palustris* | VU | [17,18] |
| REPTILIA | *Gavialis gangeticus* | CR | [17,19] |
| REPTILIA | *Ophiophagus hannah* | VU | [17] |
| REPTILIA | *Python bivittatus* | VU | [17,20] |
| AVES | *Aceros nipalensis* | VU | [21,22] |
| AVES | *Aquila clanga* | VU | [21,22] |
| AVES | *Aquila hastata* | VU | [21,22] |
| AVES | *Aquila heliaca* | VU | [21,22] |
| AVES | *Ardea insignis* | CR | [21,22] |
| AVES | *Aythya baeri* | CR | [21,22] |
| AVES | *Catreus wallichi* | VU | [21,22] |
| AVES | *Chaetornis striata* | VU | [21,22] |
| AVES | *Chrysomma altirostre* | VU | [21,22] |
| AVES | *Clangula hyemalis* | VU | [21,22] |
| AVES | *Emberiza aureola* | VU | [21,22] |
| AVES | *Falco cherrug* | EN | [21,22] |
| AVES | *Ficedula subrubra* | VU | [21,22] |
| AVES | *Francolinus gularis* | VU | [21,22] |
| AVES | *Gallinago nemoricola* | VU | [21,22] |
| AVES | *Grus antigone* | VU | [21,22] |
| AVES | *Gyps bengalensis* | CR | [21,22] |
| AVES | *Gyps tenuirostris* | CR | [21,22] |
| AVES | *Haliaeetus leucoryphus* | VU | [21,22] |
| AVES | *Houbaropsis bengalensis* | CR | [21,22] |
| AVES | *Leptoptilos dubius* | EN | [21,22] |
| AVES | *Leptoptilos javanicus* | VU | [21,22] |
| AVES | *Mulleripicus pulverulentus* | VU | [21,22] |
| AVES | *Neophron percnopterus* | EN | [21,22] |
| AVES | *Ploceus megarhynchus* | VU | [21,22] |
| AVES | *Prinia cinereocapilla* | VU | [21,22] |
| AVES | *Rynchops albicollis* | VU | [21,22] |
| AVES | *Sarcogyps calvus* | CR | [21,22] |
| AVES | *Saxicola insignis* | VU | [21,22] |
| AVES | *Sterna acuticauda* | EN | [21,22] |
| AVES | *Sypheotides indicus* | EN | [21,22] |
| AVES | *Turdoides longirostris* | VU | [21,22] |
| AMPHIBIA | *Nanorana minica* | VU | [23] |
| AMPHIBIA | *Nanorana rostandi* | VU | [24] |
| AMPHIBIA | *Scutiger nepalensis* | VU | [25] |
| ACTINOPTERYGII | *Schizothorax nepalensis* | CR | [26] |
| ACTINOPTERYGII | *Schizothorax raraensis* | CR | [26] |
| ACTINOPTERYGII | *Schizothorax richardsonii* | VU | [27–29] |
| ACTINOPTERYGII | *Tor putitora* | EN | [30] |

*VU- Vulnerable, EN-Endangered, , CR-Critically Endangered

**WORKS CITED**

1. Jnawali SR, Baral HS, Lee S, Acharya KP, Upadhyay G., Pandey M, et al. The Status of Nepal’s Mammals: The National Red List Series. Kathmandu, Nepal: Department of National Parks and Wildlife Conservation; 2011.

2. Jnawali S, Leus K, Molur S, Glatston A, Walker S. Red Panda (*Ailurus fulgens*). Population and Habitat Viability Assessment (PHVA 2010) and Species Conservation Strategy (SCS) Workshop Report. Coimbatore, India: National Trust for Nature Conservation, Kathmandu, Nepal, Conservation Breeding Specialist Group and Zoo Outreach Organization; 2012.

3. Kafle G. A review on Research and Conservation of Otters in Nepal. IUCN Otter Spec. Group Bull. 2009;26:32–43.

4. Choudhury A. Distribution and conservation of the Gaur *Bos gaurus* in the Indian Subcontinent. Mammal Rev. 2002;32:199–226.

5. Heinen JT. Population viability and management recommendations for wild water buffalo *Bubalus bubalis* in Kosi Tappu Wildlife Reserve, Nepal. Biol. Conserv. 1993;65:29–34.

6. Johnsingh AJT. Distribution and status of dhole *Cuon alpinus Pallas*, 1811 in South Asia. Mammalia. 1985;49:203–208.

7. GoN/MoFSC. The Elephant Conservation Action Plan for Nepal. Government of Nepal, Ministry of Forests and Soil Conservation/Department of National Parks and Wildlife Conservation; 2007.

8. Mitchell RM. A checklist of Nepalese mammals (excluding bats). Saugetierk Mitt. 1975;23:152–157.

9. Frick F. Die Höhenstufenverteilung der Nepalesischen Säugetiere. Säugetierkd. Mitteilungen. 1968;17:161–173.

10. Joshi AR, Garshelis DL, Smith JL. Home ranges of sloth bears in Nepal: Implications for conservation. J. Wildl. Manag. 1995;204–214.

11. Molur S, Marimuthu G, Srinivasulu C, Mistry S, et al. Status of South Asian Chiroptera: Conservation Assessment and Management Plan (CAMP) Workshop Report, 2002. Zoo Outreach Organisation and Conservation Breeding Specialist Group-South Asia in collaboration with Wildlife Information & Liaison Development Society; 2002.

12. Grassman L, Lynam A, Mohamad S, Duckworth JW, Bora JW, Wilcox D, et al. *Neofelis nebulosa*: The IUCN Red List of Threatened Species 2015: e.T14519A50656369 [Internet]. 2014 Apr. Available from: http://www.iucnredlist.org/details/14519/0

13. Smith JLD, McDougal C, Ahearn SC, Joshi A, Conforti K. Metapopulation structure of tigers in Nepal. Rid. Tiger Tiger Conserv. Hum.-Domin. Landsc. Univ. Press Camb. 1999;176–189.

14. Oli MK. Winter home range of snow leopards in Nepal. Mamm.-PARIS-. 1997;61:355–360.

15. Smith BD. 1990 Status and conservation of the Ganges River dolphin *Platanista gangetica* in the Karnali River, Nepal. Biol. Conserv. 1993;66:159–69.

16. Jnawali SR. Population ecology of greater one-horned rhinoceros (*Rhinoceros unicornis*) with particular emphasis on habitat preference, food ecology and ranging behavior of a reintroduced population in Royal Bardia National Park in lowland Nepal. [Aas, Norway]: Agriculture University; 1995.

17. Shah KB, Tiwari S. Herpetofauna of Nepal: a conservation companion. IUCN- the World Conservation Union

18. Da Silva A, Lenin J. Mugger crocodile *Crocodylus palustris*. Crocodile Status Surv. Conserv. Action Plan Third Ed Eds Manolis SC Stevenson C. 2010;94–98.

19. Maskey TM, Percival HF. Status and conservation of gharial in Nepal. Submitt. 12th Work. Meet. Crocodile Spec. Group Pattaya Thail. 1994.

20. Schleich HH, Kästle W. Amphibians and reptiles of Nepal. Biol. Syst. Field Guide Koenigstein Koeltz Sci. Books. 2002;1201.

21. Grimmett R, Inskipp C, Inskipp T. Birds of Nepal. Christopher Helm; 2000.

22. BCN, DNPWC. The State of Nepal’s Birds 2010. Bird Conservation Nepal and Department of National Parks and Wildlife Conservation; 2011.

23. Ohler A, Dutta S, Shrestha T. *Nanorana minica*: The IUCN Red List of Threatened Species 2004: e.T58432A11780511 [Internet]. 2004 Apr. Available from: http://www.iucnredlist.org/details/58432/0

24. Dutta S, Ohler A. *Nanorana rostandi*: The IUCN Red List of Threatened Species 2004: e.T58437A11781058 [Internet]. 2004 Apr. Available from: http://www.iucnredlist.org/details/58437/0

25. Ohler A, Shrestha TK. *Scutiger nepalensis*: The IUCN Red List of Threatened Species 2004: e.T57617A11664079 [Internet]. 2004 Apr. Available from: http://www.iucnredlist.org/details/57617/0

26. Terashima A. Three New Species of the Cyprinid Genus *Schizothorax* from Lake Rara, Northwestern Nepal. Jpn. J. Ichthyol. 1984;31:122–35.

27. Shrestha J. Coldwater fish and fisheries in Nepal. Fish Fish. High. Alt. Asia FAO Fish Tech Pap. 1999;13–40.

28. Petr T, Swar DB. Cold water fisheries in the trans-Himalayan countries. Food & Agriculture Org.Tech. Pap. 2002; 632 pp.

29. Shrestha OH, Edds DR. Fishes of Nepal: mapping distributions based on voucher specimens. Emporia State Res Stud. 2012;48:14–74.

30. Petr T. Fish and fisheries at higher altitudes: Asia. Food & Agriculture Org. Tech. Pap. 1999.
