# Supplementary material for: Threatened species richness along a Himalayan elevational gradient: quantifying the influences of human population density, range size, and geometric constraints
Source: BMC Ecol. 2018 Feb 7;18:6. doi: 10.1186/s12898-018-0162-3 (PMC5803900; doi:10.1186/s12898-018-0162-3)

Additional file 2: Plot S1.

**Correlation of population density, area, species range size and elevation with species richness patterns measured with empirical data and three augmentation scenarios**

Fig.1 A scatter plot showing relationship between population density and threatened species richness: (a) first augmentation, (b) second augmentation and (c) third augmentation. Regression curves with marked 95% confidence intervals were fitted by local regression method (LOESS). The dependent variable, threatened species richness, is log transformed.

a)


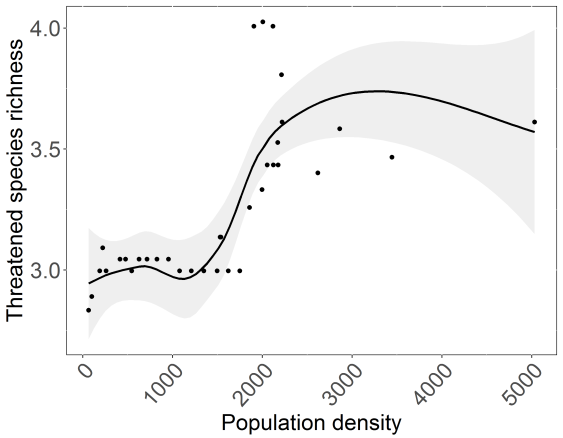


b)


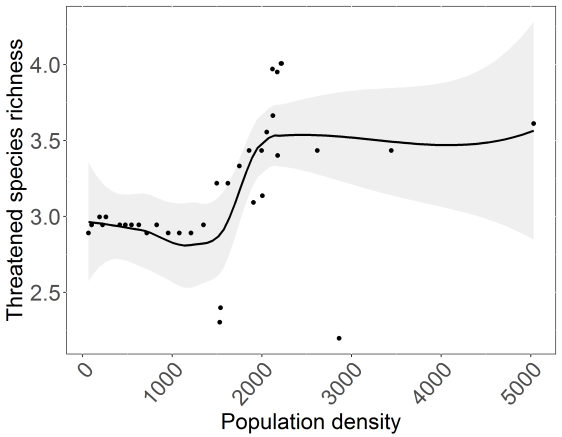


c)


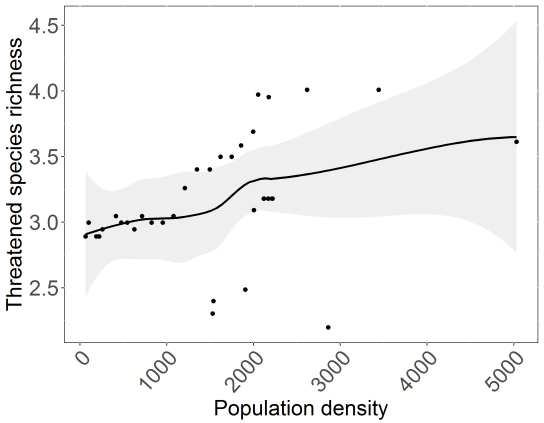


Fig.2. A scatter plot showing relationship between area and threatened species richness: (a) first augmentation, (b) second augmentation and (c) third augmentation. Regression curves with marked 95% confidence intervals were fitted by local regression method (LOESS). The dependent variable, threatened species richness, is log transformed.

a)


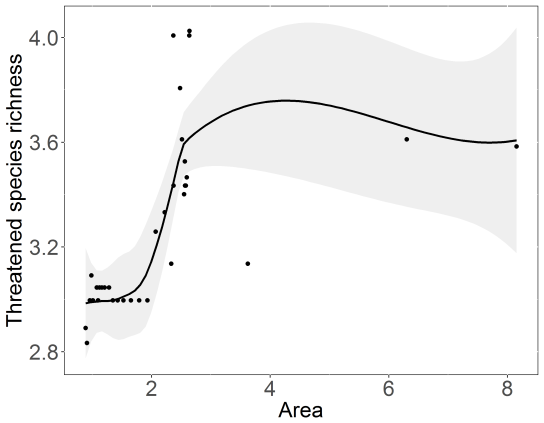


b)


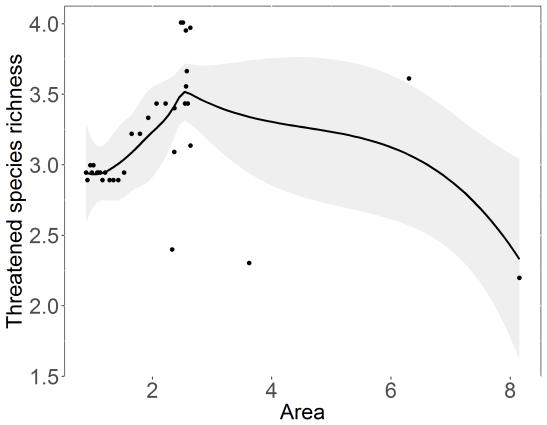


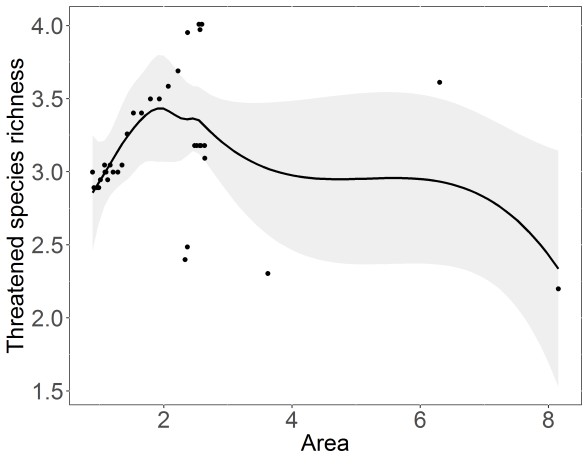


Fig.3. A scatter plot showing relationship between species range sizes and threatened species richness: (a) first augmentation, (b) second augmentation and (c) third augmentation. Regression curves with marked 95% confidence intervals were fitted by local regression method (LOESS). The dependent variable, threatened species richness, is log transformed.

a)


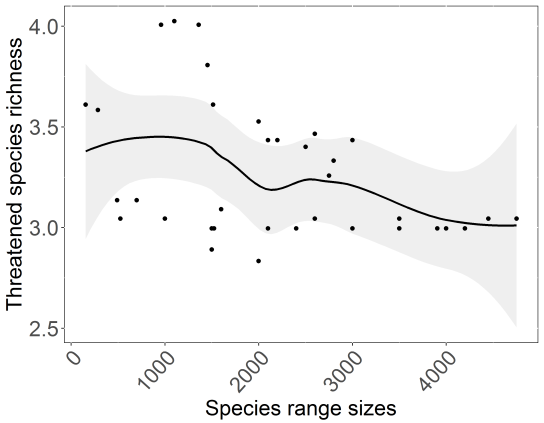


b)


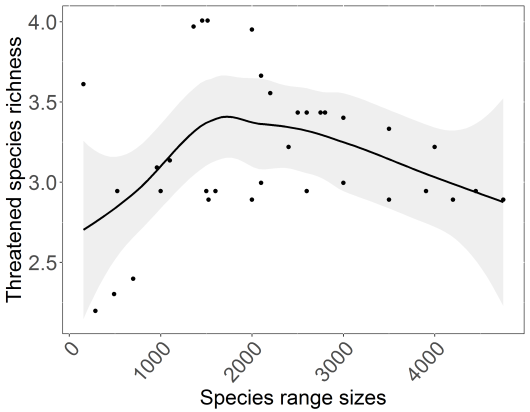


c)


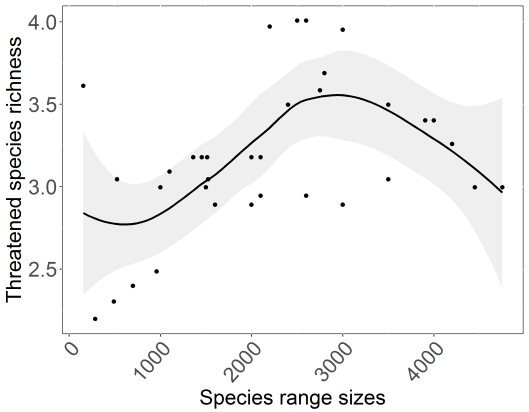


Fig.4 A scatter plot showing relationship between elevation gradient and threatened species richness: (a) first augmentation, (b) second augmentation and (c) third augmentation. Regression curves with marked 95% confidence intervals were fitted by local regression method (LOESS). The dependent variable, threatened species richness, is log transformed.

a)


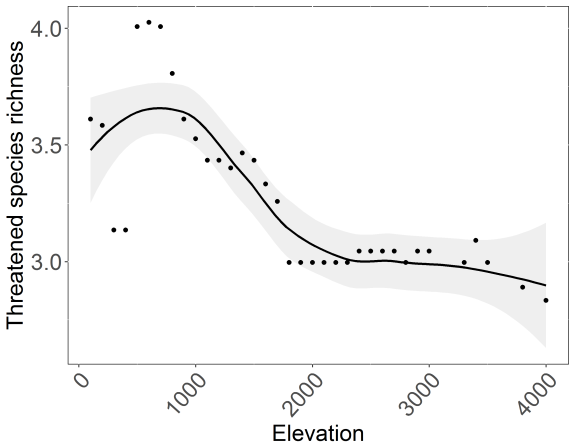


b)


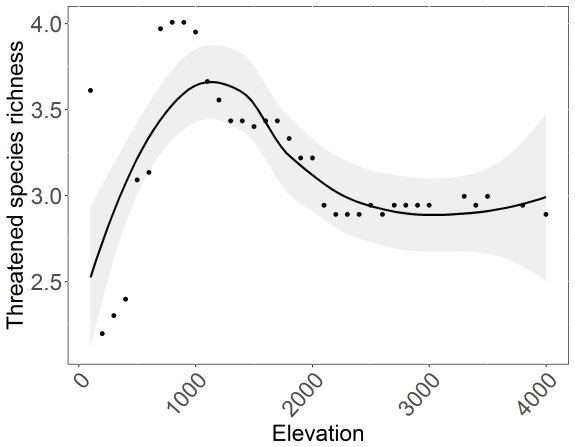


c)


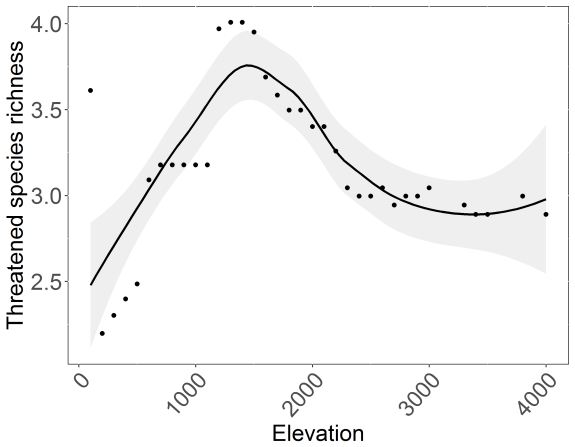

Supplement: Supplementary file 2 — Additional file 2: Plot S1. Correlation of population density, area, species range size and elevation with species richness patterns measured with empirical data and three augmentation scenarios. [file 12898_2018_162_MOESM2_ESM.docx]
